# How single-cell techniques help us look into lung cancer heterogeneity and immunotherapy

**DOI:** 10.3389/fimmu.2023.1238454

**Published:** 2023-08-21

**Authors:** Pu Liao, Qi Huang, Jiwei Zhang, Yuan Su, Rui Xiao, Shengquan Luo, Zengbao Wu, Liping Zhu, Jiansha Li, Qinghua Hu

**Affiliations:** ^1^ Department of Pathology, Union Hospital, Tongji Medical College, Huazhong University of Science and Technology, Wuhan, Hubei, China; ^2^ Key Laboratory of Pulmonary Diseases of Ministry of Health, Tongji Medical College, Huazhong University of Science and Technology, Wuhan, China; ^3^ Department of Respiratory and Critical Care Medicine, Hubei Province Clinical Research Center for Major Respiratory Diseases, National Health Commission (NHC) Key Laboratory of Pulmonary Diseases, Union Hospital, Tongji Medical College, Huazhong University of Science and Technology, Wuhan, Hubei, China; ^4^ Department of Pathophysiology, School of Basic Medicine; Tongji Medical College, Huazhong University of Science and Technology, Wuhan, China; ^5^ Department of Neurosurgery, Tongji Hospital, Tongji Medical College, Huazhong University of Science and Technology, Wuhan, Hubei, China; ^6^ Institute of Pathology, Tongji Hospital, Tongji Medical College, Huazhong University of Science and Technology, Wuhan, Hubei, China

**Keywords:** single-cell analysis, lung cancer, immunotherapy, heterogeneity, tumor microenvironment

## Abstract

Lung cancer patients tend to have strong intratumoral and intertumoral heterogeneity and complex tumor microenvironment, which are major contributors to the efficacy of and drug resistance to immunotherapy. From a new perspective, single-cell techniques offer an innovative way to look at the intricate cellular interactions between tumors and the immune system and help us gain insights into lung cancer and its response to immunotherapy. This article reviews the application of single-cell techniques in lung cancer, with focuses directed on the heterogeneity of lung cancer and the efficacy of immunotherapy. This review provides both theoretical and experimental information for the future development of immunotherapy and personalized treatment for the management of lung cancer.

## 1 Introduction

### 1.1 Tumor heterogeneity is an unmet challenge in the immunotherapy of lung cancer

The advent of immune checkpoint inhibitors (ICIs) over the past decade has ushered in an rapid-growth era of immunotherapy. At present, ICIs used in clinical practice mainly include monoclonal antibodies against programmed death ligand 1 (PD-L1), programmed death receptor 1 (PD-1) and cytotoxic T lymphocyte-associated protein 4 (CTLA 4). With its unique mechanism of action and excellent clinical efficacy, it represents a revolution in tumor treatment following surgery, radiotherapy, chemotherapy and targeted treatment for a variety of malignancies (1). Lung cancer is currently the deadliest malignancy across the globe (2). Thanks to the antibodies targeting PD-1 or PD-L1, the overall survival in patients with advanced non-small cell lung cancer (NSCLC) has been significantly improved, and the five-year survival in PD-L1-positive patients has been raised from no more than 5% to virtually 30% (3, 4). Integration of PD-L1 inhibitors into the first-line platinum-based chemotherapy could enhance survival rate in patients with widespread small cell lung cancer (SCLC) (5). Nonetheless, lung cancer is a highly heterogeneous tumor and studies showed that the heterogeneity of tumor microenvironment (TME) mediates cancer progression and response to immune checkpoint inhibitors (ICI) (6, 7). The current development of immunotherapies for lung cancer has been hampered by the lack of biomarkers predictive of efficacy, and the lack of more immunotherapeutic targets, and lower remission rate (8). Hence, a comprehensive look at the lung cancer ecosystem is warranted in order to improve personalized immunotherapies.

The ecosystem of lung cancer consists of cancer cells, immune cells, stromal cells, non- cellular tissue components, among others. Their interactions dictate the disease progression and the response to treatment (9, 10). Heterogeneity of tumor ecosystem is an important factor that renders tumor therapy difficult, and the genes and morphology related to tumor heterogeneity depend on the intricate interaction between genetic factors and environment (11, 12). Extensive phenotypic and genetic variations exist not only among tumor patients (heterogeneity between tumors), but also within a single tumor (heterogeneity within tumors), including spatial heterogeneity (different genotypes and phenotypes are found in different regions of the same tumor) and temporal heterogeneity (genes and phenotypes differ in primary and secondary tumors). Tumor heterogeneity leads to diversity in cancer signaling pathways and variation in cancer phenotypes, presenting a major challenge for personalized cancer treatment (12).

The molecular heterogeneity of lung cancer (including the differences among and within tumors) has become a subject of active investigations of lung cancer immunotherapy. The heterogeneity includes but is not limited to the molecular expression heterogeneity of tumor and immune cells, especially the heterogeneity of genetic phenotypes and antigen presentation molecule expression etc. (13, 14). The rapid development of single-cell techniques has allowed for the determination of the heterogeneity and immune microenvironment of lung cancer cells and other cell types (15–17). These techniques can help us gain insights into the development and progression of lung cancer, and the complicated mechanism of immunotherapy, thereby improving immunotherapeutic strategies.

### 1.2 Technical advantages of the single-cell technology over traditional bulk sequencing

Conventional bulk transcriptome and genome analyses have substantially contributed to our understanding of tumor evolution and growth. Whereas, signals displayed by some particular group or state of cells will be masked in the process of bulk sequencing, and such specific cell populations or states are sometimes very critical, such as tumor stem cells and infiltrating immune cells that are related to the tumor response to treatment. Therefore, examining individual cells at the genomic, transcriptomic, epigenomic and proteomic levels can help us better understand tumor heterogeneity at molecular levels and overcome the limitations of the traditional bulk sequencing and allows for high-granularity analyses at cellular and molecular levels (18, 19). This feature has a good prospect of application in the field of tumor immunotherapy, since single-cell analysis can identify cell pathways and types involved in tumor response and immune escape.

The current single-cell technology involves a series of rapidly developing methodologies. The most commonly used single-cell technologies for tumor immunotherapy include single-cell RNA sequencing for transcriptomic analysis, mass spectrometry flow cytometry for proteomic analysis, and spatial molecular analysis (20–22). Each of these techniques delineates a high-dimensional molecular contour for a single cell, which can be classified, by calculation, into different cell groups. For instance, the results of these techniques are more representative than typical cell type markers. Meanwhile, the high-dimensional characteristics of these methods can more accurately describe cell types and infer the relationship among molecular pathways and transition of cell status (21). These characterizations identify the pathways underlying the behavior of each different cell type through complementary computational techniques, and infer the intercellular and intracellular interactions associated with cell state transitions. Therefore, the inference of those pathways mirrors the ongoing clinical research effort in anti-tumor immunotherapy, and the exact medical strategies being developed to reconnect TME using combination therapies to achieve immunotherapy sensitization.

### 1.3 How to combine single-cell analysis with immunotherapy for lung cancer?

**Figure 1:**
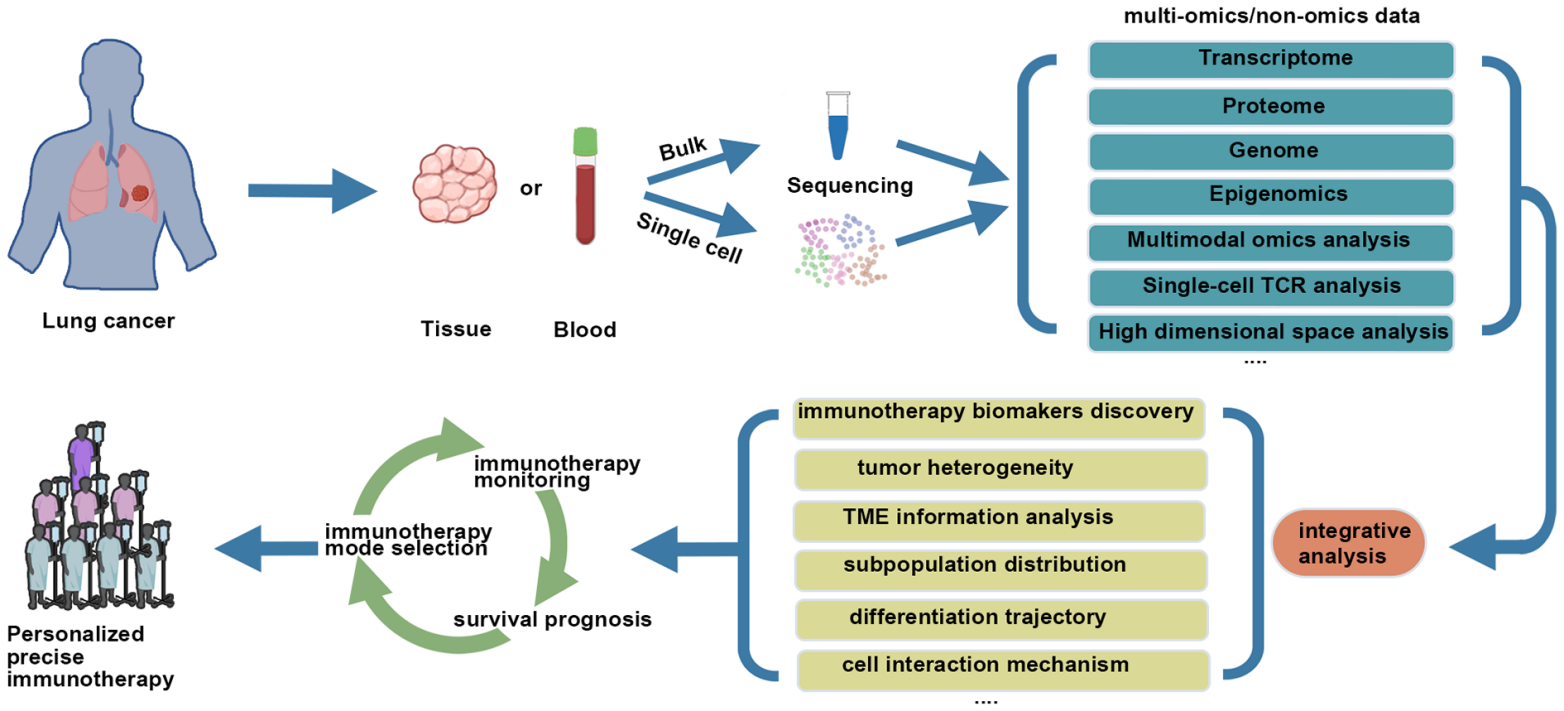
Combining single cell analysis with immunotherapy for lung cancer.

(**Figure 1**) First of all, single-cell omics analysis is performed on tumor tissues from lung cancer patients, and appropriate single-cell analysis strategies are selected according to the purpose of the study. It is desirable if peripheral blood samples of corresponding patients are used to monitor immune indicators. Secondly, components of TME are analyzed based on single-cell omics data, with focus being directed at tumor cell heterogeneity, the subtype and status of immune cells, then tumor immunity-related indicators or signatures are established. Finally, appropriate data sets regarding immunotherapeutic strategies are used for further clinical verification of treatment mode selection and monitoring of response, prognosis and other aspects of lung cancer patients.

(**Table 1**) Here, we first investigated, in general, how single-cell analysis has been used for the study of the interaction between lung cancer cells and TME, and, in particular, how it is related to the response to anti-tumor immunotherapy. Then, we examined the role of single-cell TCR analysis in immune oncology. After this, we looked at the emerging technologies for single-cell spatial analysis, especially their utility to immune oncology. Finally, we discussed the future trend of single-cell technology and its potential role in promoting the application of immune oncology in lung cancer.

**Table 1:** Applications of single-cell analyses in lung cancer immuno-oncology.

PMID/Refs.	Author	Single-cell technologies used	lung cancer type	Cell types characterized	Clinical/cellular/animal level	Key findings
33144684 (23)	He D et al.	scRNA-seq	EGFR mutant LUAD	tumor cells	clinical	ELF3 is upregulated in tumor cells under the secretion of immunoinfiltrating inflammatory cytokines, which activates the PI3K/Akt/NF-κB pathway and up-regulates the expression of proliferative and anti-apoptotic genes.
30821712 (24)	Ma KY et al.	scRNA-seq	LUAD	tumor cells	cellular	Miscoordinated expression of IFN-γ signaling pathway and lower expression of MHC II gene, MHC II and IFN-γ signaling pathway jointly determine the formation of immunotherapy resistance.
36195615 (25)	Tian Y et al.	scRNA-seq	SCLC	tumor cells, T cells, macrophages	clinical	1.Patients with weak immune characteristics SCLC are more likely to benefit from immune checkpoint block (ICB) than those with strong immune characteristics SCLC.
						2. Established a detailed immune map of SCLC.
						3. The detailed classification of T cells in SCLC also revealed the expression pattern of dysfunction and exhaustion markers (such as PDCD1, CTLA4, HAVCR2, LAG3, TIGIT and LAYN), which may be used as immunotherapeutic targets.
						4. Non neuroendocrine SCLC subtype cells tend to have more interactions with other cells and immune and stromal cells, and may be related to the clinical results of immunotherapy.
34653364 (26)	Chan JM et al.	scRNA-seq	SCLC	tumor cells	clinical	PLCG2 overexpression subsets were associated with metastasis, immunosuppression and poor prognosis.
29942094 (27)	Guo X et al.	scRNA-seq, scTCR-seq	NSCLC	T cells	clinical	1. Mapped the immune map of lung cancer T cells at the single cell level.
						2. Higher rates of “pre-exhausted” and exhausted CD8 T cells are associated with a better prognosis for lung adenocarcinoma, the proportion of activated Treg cells is negatively correlated with the prognosis of lung adenocarcinoma patients.
						3. The state transition of CD8+T cell cluster in NSCLC was deduced by scTCR-Seq.
34099454 (28)	Zhang Y et al.	scRNA-seq	NSCLC	T cells	clinical	In immunotherapy resistant patients with MET gene amplification, the proportion of XTIST/CD96/KLRG1 triple positive NK cell subpopulation increased and the proportion of CD8+T cells and NK cell subpopulations decreased.
33506299 (29)	Zhong R et al.	scRNA-seq	NSCLC	T cells	clinical	The change in the percentage of NK cells and T cells may be related to the effective treatment of pabolizumab.
33777802 (30)	Liu S et al.	scRNA-seq	NSCLC	T cells, macrophages	clinical	CD8+T cells, INF-γ+CD8+T cells and the ratio of M2/M1 like macrophages was related to the subsequent better immunotherapy results in patients receiving EGFR-TKI treatment.
35140113 (31)	Yang L et al.	scRNA-seq	EGFR mutant LUAD	T cells	clinical	EGFR mutated tumor cells secrete cytokines to recruit various immunosuppressive cells, while activated immune cells (CD8+TRM and CXCL9+TAM) are seriously insufficient.
30979687 (32)	Zilionis et al	scRNA-seq	NSCLC	TIMs	clinical	Found 25 TIMs states, which may become a new target for immunotherapy.
28475900 (33)	Lavin et al.	scRNA-seq, CyTOF	LUAD	macrophages, T cells	clinical	1. Obtained the characteristic genes of tumor infiltrating macrophages such as TREM2, CD81, MARCO, APOE, etc.
						2. The immune cells in the microenvironment of early lung adenocarcinoma were mapped in detail, and T cells were divided into 21 subgroups with different Marker expression patterns.
						3. The content of Treg cells in early tumor stage patients was significantly increased, and it grew rapidly in early tumor stage, and PD-1 was significantly expressed in CD4+and CD8+cells in tumor tissue.
32385277 (34)	Kim N et al.	scRNA-seq	LUAD	vascular endothelial cells	clinical	Vascular endothelial cells in LUAD reduced their antigen presentation and homing activity of immune cells through remodeling, thereby promoting tumor immune tolerance.
33953163 (35)	Wu F et al.	scRNA-seq	NSCLC	macrophages	clinical	Macrophages play a major role in inhibiting T cell function through checkpoint pathway.
31811131 (36)	Karacosta LG et al.	CyTOF	NSCLC	tumor cells	cellular	The increased expression of PD-L1 during EMT confirms that EMT is associated with tumor immune escape.
31957112 (37)	Shaul ME et al.	CyTOF	NSCLC and SCLC	neutrophils	clinical	Three main subtypes of LDN/HDN have immune characteristics and inherent plasticity.
336725934 (38)	Sorin M et al.	IMC	LUAD	Neutrophils, monocyte, T cells, macrophage	clinical	1. Tissue relationship of cells in the immune microenvironment is of uniquely prognostic value.
						2. The increased proportion of HIF1α+ neutrophils subgroup was significantly correlated with poor overall survival.
36725085 (39)	Sorin M et al.	IMC	NSCLC	tumor cells, T cells,monocyte	clinical	The expression of CXCL13 was related to the ICI efficacy, and the recombinant CXCL13 enhanced the response to anti-PD-1 *in vivo*, which could be ascribed to increased T-cell subpopulations subjected to antigen stimulation and decreased CCR2+ monocytes.
34767762 (40)	Leader AM et al.	scRNA-seq, CITE-seq, TCR-seq	NSCLC	tumor cells, T cells, plasma cells, macrophages	clinical	Constructed the immunoreactive cell atlas of early lung cancer, and established the LCAM module, which can be used as a more direct indicator of antigen-specific anti-tumor immune activation.
35452604 (41)	Hanada KI et al.	CITE-seq, TCR-seq	NSCLC	TILs	clinical	A molecular label of neoantigen reactive T cells based on CD39 protein and CXCL13 mRNA expression was defined to rapidly and efficiently identify CD4+ and CD8+T cells with neoantigen reactive TCRs.
35331733 (42)	Ma Y et al.	scTCR-Seq	NSCLC	TILs	clinical	Tumor specific TCRS were identified, and the corresponding TCR-T cells can specifically recognize and kill autologous tumor cells, which can be used for personalized immunotherapy in advanced cancer patients.
33064988 (43)	Ott PA et al.	scTCR-Seq	NSCLC	T cells	clinical	Revealed the dynamic changes of the clonal type of tumor neoantigen vaccine-specific T cells, and proved that the T cell immune response induced by tumor new antigen vaccine was highly specific and effective.
35831283 (44)	Hui Z et al.	scRNA-seq, scTCR-Seq	NSCLC	T cells	clinical	TNFRSF4 can be used as a potential target to reduce the function of Treg and improve the anti-tumor immunity to NSCLC.
32591861 (45)	Zhang F et al.	scRNA-seq, scTCR-Seq	NSCLC	T cells	clinical	1. Tumor-related CD4+T cell clones had higher cytotoxicity than CD8+T cell clones.
						2. After lung cancer progression, the abundance of tumor-related CD4+T cell clones decreased significantly, and the percentage of PD-1+T cells decreased.
						3. The pseudo-time track of CD8+T cell clone corresponds to the immunotherapy time point, indicating that the activity of the “cytokine and cytokine receptor interaction” pathway decreased.
33514641 (46)	Gueguen P et al.	scRNA-seq, scTCR-Seq	NSCLC	TILs	clinical	The differentiation of these two CD8+TIL subpopulations from precursor to late stage is related to TCR amplification and T-cell cycle in tumor.
36434043 (47)	Zhu J et al.	scRNA-seq, spatial transcriptome	LUAD	tumor cells, Treg	clinical	The spatial changes of TGF-β signaling pathway in the interaction between cancer cells and TME and in the regulation of immune escape in the invasion of LUAD.
33972311 (47)	Sinjab et al.	scRNA-seq, spatial transcriptome	LUAD	tumor cells, dendritic cells, macrophages	clinical	1. The overlap of immune checkpoint-receptor and cytokine receptor (L-R) interactions between LUAD tumor-distal regions was reduced compared with L-R interactions between LUAD tumor-proximal regions.
						2.The interaction between the immune checkpoint proteins CD24 and LGALS9 in tumor epithelial cells, SIGLEC10 in dendritic cells, SIGLEC10 and HAVCR2 in macrophages increased.
32253229 (48)	Zugazagoutia J et al.	DSP, mIF	NSCLC	tumor cells, T cells	clinical	The high level of CD56 expression in the immune cell region (CD45+) was associated with longer PFS and OS in NSCLC patients receiving PD-1 checkpoint inhibitor monotherapy.
32253229 (49)	Moutafi MK et al.	DSP	NSCLC	tumor cells	clinical	The expression of CD44 in tumor cells is closely related to the prolonged PFS and OS, which can be used as an independent evaluation factor to predict the clinical benefits of patients receiving PD-1 inhibitor treatment.

scRNA-seq, single-cell RNA sequencing; scTCR-seq, single-cell T cell receptor sequencing; CyTOF, mass spectrometry flow cytometry; IMC, imaging mass cytometry; CITE-seq, cellular indexing of transcriptomes and epitopes by sequencing; DSP, digital spatial profiling; mIF, multiplexed immunofluorescence; EGFR, epidermal growth factor receptor; LUAD, lung adenocarcinoma; SCLC, small cell lung cancer; NSCLC, non small cell lung cancer; TIMs, tumor infiltrating myeloid cells; TILs, tumor infiltrating lymphocytes; Treg, regulatory T cells.

## 2 Single-cell omics in lung cancer

### 2.1 Transcriptome

Single-cell RNA sequencing (scRNA-seq) is a non-targeted technology for the quantification of transcripts in a single cell, and is often used to identify new cell types, find rare cell populations, and construct maps of cell status and phylogeny (50–52). scRNA-seq can help us gain insights into the distribution, status, action process and cooperation mechanism of different subpopulations of similar cells, and, from a new perspective, look at the heterogeneity of lung cancer, and the interaction between lung cancer cells and TME, especially their relationship with anti-tumor immunotherapy response (15–17). Due to the rapid technical development of scRNA-seq and cell separation, the number of cells sequenced has grown from hundreds to thousands, and the technique is becoming increasingly cost-effective. The analytical methods are also improving constantly, covering determination of cell types, dimensionality reduction of high-dimensional data, unsupervised clustering, phylogenetic modeling, trajectory inference, RNA velocity analysis, and collaborative analysis of multiple data sets (53–57).

#### 2.1.1 Tumor cells and immunotherapy responses

The main feature of lung cancer revealed by scRNA-seq is intratumoral and intertumoral heterogeneity. The scRNA-seq can make more precise diagnosis and prognostic predictions, and facilitate the development of new anti-lung cancer agents. For example, Wu F et al. (35) utilized scRNA-seq and analyzed 42 samples from patients with advanced NSCLC at various stages. They found that the intertumoral and intratumoral heterogeneity of lung adenocarcinoma (LUAD) was lower than that of lung squamous cell carcinoma (LUSC). In addition, cancer cells from different patients exhibited higher heterogeneity. Next, the researchers used scRNA-seq data to infer copy number changes (CNAs) in the cancer cell population and to reveal heterogeneity between and within patients. Most patients, especially LUAD patients, had dominant clones, while in a few LUSCs, malignant cells were distributed in multiple clusters. To quantify intratumoral heterogeneity, they obtained expression-based and CNA-based intratumoral heterogeneity scores (ITH), which were designated ITH_GEX_ and ITH_CNA_, respectively. The patients were further divided into three categories in terms of lung cancer types and mutations: LUAD patients with driving mutations (LUADm), LUAD patients without driving mutations (LUADn) and LUSC patients without driving mutations (LUSCn). Their results showed that, compared with LUADm patients, LUSCn patients had significantly higher ITH_CNA_, but no significant difference was found in ITHGEX. ScRNA-seq demonstrated a transcriptional heterogeneity within the malignant cell population, which was associated with driving mutations. Identification of alterations in more diverse subpopulations may have implications for immunotherapy.

It is also feasible to use scRNA-seq to identify ubiquitous tumor cells with specific transcriptomic status in lung cancer patients, which helps us better understand the tumor type and cell hierarchy of lung cancer, and single out transcriptome signature related to the response and resistance to treatment (23, 58). Mounting evidence shows that EGFR mutation is an important factor affecting the therapeutic efficacy of PD-1 inhibitors in NSCLC patients, and patients with EGFR mutation responded to the treatment less well than their counterparts without EGFR mutation (59–62). He D et al. (23), by employing scRNA-seq, revealed a significant heterogeneity in EGFR mutation in patient with early-stage LUAD, and found that ELF3 was one of the most up-regulated genes in advanced tumor cells. Under the effect of immune infiltrating inflammatory cytokines (such as IL1B), ELF3 in tumor cells was up-regulated, thereby activating PI3K/Akt/NF- κB pathway, and up-regulating the expression of proliferation and anti-apoptosis genes, such as BCL2L1 and CCND1. These findings suggested that there existed an involved interaction among tumor cells, stromal cells and immune infiltration cells in TME. These results may pave the way to immunotherapy targeting EGFR mutant LUAD.

Meanwhile, scRNA-seq analysis has multiple advantages in that it not only can reveal the molecular diversity of different samples and show the impact of clinical treatment on different cell subsets. Ke-Yue Ma, et al. (24) utilized scRNA-seq to examine the heterogeneity of genes associated with response of LUAD to immunotherapy. They compared LC2/ad (Vandetanib sensitive) and LC2/ad-R (Vandetanib tolerant) cell lines, and found that LC2/ad had a higher level of MHC II gene and IFN-γ signal pathway coexpression gene. However, the IFN-γ signaling pathway in LC2/ad-R was down-regulated and the expression of MHCII gene was low. They revealed a possible mechanism of Vandetanib resistance: that is, MHC II and IFN-γ signaling pathways jointly determined the development of immunotherapy resistance.

Tian Y, et al. (25), by using scRNA-seq, examined about 5000 matched normal adjacent tissues (NAT) and primary tumors (PT) cells from 11 SCLC patients (including a patient with both primary tumor (PT) and recurrent tumor (RT)). They found that human SCLC had a significant inter-tumor and intra-tumor heterogeneity, and many tumors contained separate subpopulations, indicating there is a remarkable intra-tumor heterogeneity at the transcriptomic level. In addition, most SCLCs with neuroendocrine (NE) characteristics, such as SCLC-N and SCLC-A, tended to have strong immunological features, while non-NE SCLCs, such as SCLC-P and SCSC-Y, tended to possess weak immune traits. Patients with SCLC having weak immune features were more likely to benefit from immune checkpoint blockade (ICB) than their counterparts with strong immune characteristics. The scRNA-seq data revealed that multiple SCLC subtypes showed different proportions in practically all SCLC patients, highlighting the importance of scRNA-seq and the need for functional research on tumor progression and immunotherapy of ITH. Moreover, Chan JM et al. (26) applied scRNA-seq to analyze the transcriptome of 21 fresh SCLC samples from 19 patients and 155098 cells from 24 LUAD samples and 4 normal lung tissue samples from the area adjacent to cancer. They found that the level of copy number variation (CNV) was higher in SCLC than in LUAD and had significant heterogeneity. They exhibited that PLCG2 overexpression subsets were associated with metastasis, immunosuppression and poor prognosis. Therefore, it is potentially of great significance for the design of novel strategies of the targeted therapy and immunotherapy. These observations collectively demonstrated that ScRNA-seq can help researchers better understand tumor heterogeneity and the intricate interactions between tumor cells and their microenvironment, thereby facilitating the identification of lung cancer cell subpopulations amenable to immunotherapy.

#### 2.1.2 Immune, stromal cells, and immunotherapeutic responses

Infiltrating immune cells, cancer-associated fibroblasts and vascular endothelial cells are important components of TME (63). By means of scRNA-seq, we can identify the features of various types of immune, stromal cells, heterogeneous expression profiles, and look into mechanisms involved in immunosuppression, thus better understanding the heterogeneity and diversity of cancer immune responses.

In view of the intricacies of the TME, in recent years, single-cell transcriptome sequencing has been incrementally used in the study of infiltrating immune cells in lung cancer (25, 27–31, 45). It has an important implication for the research of the mechanism underlying the lung cancer tumor immunity, especially for the study of the functional status of T cells in the tumor, which play a pivotal part in killing tumor cells, for the development of efficacious immunotherapy and the identification of sensitive targets and markers. Guo X et al. (27) conducted scRNA-seq on 12346 T cells from the peripheral blood, cancer-adjacent tissues and cancer tissues of 14 NSCLC patients prior to drug treatment, worked out the immune map of lung cancer T cells at the single-cell level, revealed the heterogeneity of lung cancer T cells, and provided a new notion for immunotherapy to specifically target T cell subsets. This study identified 16 major clusters of T cells (7 CD8 and 9 CD4 types). In addition to exhausted CD8 T cells, the infiltrating CD8 T cell population of lung cancer was also found to include two groups of “pre-exhausted” CD8 T cells that may bear a state transition relationship with exhausted CD8 T cells. Moreover, higher ratios of “pre-exhausted” and exhausted CD8 T cells were associated with a more favorable prognosis for lung adenocarcinoma. Apart from that, in terms of the expression of TNFRSF9 (4-1BB), a group of activated Tregs could be distinguished from lung cancer-infiltrating regulatory T cells (Tregs). The expression of inhibition-related genes in this group of Tregs was higher, suggesting that they were the Treg cells that actually serve the inhibition function in tumors. At the same time, the proportion of activated Treg cells was negatively correlated with the prognosis of lung adenocarcinoma.

In NSCLC patients receiving ICI, Zhang Y et al. (28) found that patients with MET gene amplification were refractory to the treatment. After analysis of more than 20000 immune cells with scRNA-seq, the researchers identified a new XTIST/CD96/KLRG1 triple positive NK cell subpopulation in patients with MET amplification. In immunotherapy-resistant patients, the proportion of this subpopulation was elevated and the proportion of NK cells and CD8+T cell subpopulations dropped. Moreover, some researchers used scRNA-seq to study the dynamic change of peripheral blood T cell clones in NSCLC patients receiving PD-1 inhibitors (45), and found that the number of a CD4+T cell clone related to tumor significantly dropped upon tumor progression, and the proportion of PD-1+T cells also decreased significantly. What’s more, an NSCLC patient with negative PD-L1 expression benefited from the treatment of pabolizumab (PD-1 inhibitor) (29). An scRNA-seq analysis of the patient’s peripheral blood revealed that the NKG7+NK cells and NKG7+T (NKT) cells of the patient were significantly lowered, while the CD8+T cells and Naive T cells were prominently increased, suggesting that the change in the percentage of NK cells and T cells might be related to the efficacy of pabolizumab treatment.

Liu S et al. (30) examined whether the patient’s previous response to EGFR-TKI was related to the subsequent immunotherapy results. They found that, in patients receiving TKI (Tyrosine Kinase Inhibitor) treatment, the objective response rate (ORR) of immunotherapy was significantly higher in patients with short progression free survival (PFS) than in those with long PFS. By comparing the TME of the two groups using scRNA-seq, the researchers found that the infiltration rate of INF-γ+CD8+T cells and CD8+T cells in the immune microenvironment was higher in patients with short PFS, and the ratio between M2- and M1-like macrophages was significantly lower in short-PFS patients than in their counterparts with long PFS. Therefore, this study provided a marker reference from the angle of a single cell for the ensuing treatment in patients who had received EGFR-TKI targeted therapy. scRNA-seq analysis by Yang L et al. (31) revealed that the TME of EGFR mutant LUAD and wild type LUAD had different heterogeneity in cell composition, function and their interaction. The loss of proinflammatory cells, enrichment of inhibitory cell types and the low expression of immune checkpoint proteins may lead to an immune silence environment for EGFR-mutated LUAD, i.e., EGFR-mutated tumor cells secrete cytokines to recruit various immunosuppressive cells, while activated immune cells (CXCL9+TAM and CD8+TRM) were seriously insufficient. Therefore, in future, effective immunotherapy can be accomplished in EGFR mutant LUAD patients by improving the inhibitory tumor immune microenvironment (TIME).

Tian Y, et al. (25) employed scRNA-seq to develop a detailed immunity map of SCLC. Compared with normal adjacent tissues, the proportion of lymphocytes in primary SCLC was higher and the proportion of myeloid cells lower, indicating that adaptive immunity in TME played a more important role. Then, the researchers re-classified T cells and myeloid cells and categorized macrophage into four groups: a tumor-associated macrophage groups and three groups of resident alveolar macrophages. T cells from normal adjacent tissues and TME were predominantly CD8+ T cells and highly expressed cytotoxic markers, suggesting that immunological assessment of SCLC is of great significance. Moreover, reclassification of T cells in SCLC patients can better mirror the expression pattern of T cells dysfunction and exhaustion markers (such as HAVCR2, CTLA4, LAYN, PDCD1, LAG3, TIGIT), which may be used as immunotherapeutic targets. scRNA-seq of T cell subsets of TME in SCLC revealed that HAVCR2 had the highest expression level in exhausted T cell subsets, while LAYN was sporadically expressed in exhausted CD8 + T cells, and CTLA4 was preferentially expressed on other T cells. T cell heterogeneity and co-inhibitory receptor expression preference in SCLC patients provide potential immunotherapeutic targets.

Tumor-infiltrating myeloid cells (TIMs), such as monocytes, macrophages, dendritic cells and neutrophils, have been identified as a key regulator of cancer growth (64, 65). Zilionis et al. (32) used scRNA-seq to locate TIMs in NSCLC patients and identified 25 TIMs states, most of which could be found repeatedly in patients. This study provides a new theoretical basis for future elucidation of the role of myeloid cells in cancer, and TIMs may serve as a new target for immunotherapy. Lavin et al. (33) conducted scRNA-seq to analyze the TME of 18 LUAD patients, and identified the characteristic genes of tumor infiltrating macrophages, such as TREM2, CD81, MARCO, APOE, etc. In addition, analysis with scRNA-seq found that vascular endothelial cells from NSCLC patients reduced their antigen presentation and the homing activity of immune cells through remodeling, thereby promoting tumor immune tolerance (34, 66). A subgroup of cancer-associated fibroblasts (CAFs) that highly expressed extracellular matrix protein genes were found to induce immunotherapy resistance by increasing the levels of PD-L1 and CTLA-4 proteins in Treg cells through cell cross talk (67).

Overall, a comprehensive understanding of the composition and state of immune cells is crucial to the elucidation of the responsiveness and resistance to current immunotherapies, and to the design of new immunomodulatory therapies. Use of the single-cell transcriptomic analysis in lung cancer patients receiving immunotherapy also revealed the heterogeneity and diversity of lung cancer immune responses. Clusters based on non-targeted transcriptional assessment of cell state often cannot be completely separated into traditional immune cell subpopulations in terms of cell surface protein expression. For immunotherapy, application of these methods to immune cells and stromal cells in TME can help elucidate the response to immunotherapy (such as ICIs) and the transcriptional state of drug-resistant cells. Importantly, the analysis aimed at mapping the immune picture of multiple lung cancer types has identified multiple new transcriptional states, which are related to the responsiveness of lymphocytes, DC cells, monocytes, macrophages and fibroblast compartment of TME to immunotherapy. Researchers can now look into how lung cancer and the immune system co-evolve during treatment and recurrence at the single-cell level.

#### 2.1.3 Cell interaction analysis

Very complex interactions take place between tumor or immune cells and/or stromal cells, which together dictate the tumor progression and response to treatment. This cell-cell interaction can be studied by calculating the expression level of receptor and ligand from scRNA-seq data. At the same time, for the exploration of the complex interaction between tumor cells and TME, new technologies, including single-cell space transcriptome methods, are also developing rapidly, making it possible to look into the cell-cell physical interaction. At present, the extensively used cell interaction analysis algorithms include CellPhoneDB, CellChat, iTALK, NicheNet, among others (68–71), which is out of the scope of the review.

To look into the interaction between different cell types in TME, Wu F et al. (35) examined the cell-cell interaction by using scRNA-seq to get a full picture of TME in NSCLC patients, including angiogenesis, T cell activation, CAF activation, immunosuppressive cell recruitment, and activation of checkpoint routes. Obvious interactions were found between cancer cells and fibroblasts, endothelial cells and macrophages. An analysis of the cross cell interaction molecules revealed a complex network involving multiple carcinogenic and inflammatory signaling pathways. The researchers found that, in patients’ immune environment, macrophages played a key role in the inhibition of T cells *via* checkpoint pathway. Furthermore, predominant pathways vary with different subgroups of NSCLC. For instance, LUAD driving gene mutation had a high level of TIGIT pathway activation, but a low level of TIM3 (HAVCR2) pathway activation. Except in few LUSC patients, the authors did not detect any significant activation of PD1/PD-L1 axis, which might be ascribed to the low expression of PD1/PD-L1 at the transcriptomic level. Even in the same subtype of lung cancer, the interactions related to immunotherapy were different, highlighting the need for more precise biomarkers to improve the efficacy of immunotherapy. In addition, Tian Y, et al. (25) used CellPhoneDB to study the interaction between ligand-receptor pairs, and confirmed that the non-neuroendocrine small cell lung cancer subtypes (SCLC-non-NE) interacted more with other cells, including immune and stromal cells than other subtypes of SCLC, and may be related to the clinical outcomes of immunotherapy. By correlating the expression of ligand receptor pairs between different cell types, important information on cell-to-cell interactions related to lung cancer immunotherapy can be inferred from scRNA seq data, which may help us better understand the complicated arrangement and interaction between tumor cells and TME, as well as to find new indicators or signatures related to lung cancer immunotherapy.

#### 2.1.4 Limitations of scRNA-seq

ScRNA-seq is the most widely used single-cell sequencing technique. With its extensive application in scientific research, scRNA-seq gradually some methodologically inherent problems began to emerge. First, efficiency of mRNA capture in the single-cell transcriptome method is low, standing at somewhere between 5% and 15%, leading to data sparsity, sampling deviation, and loss of low-level transcript gene expressions (72). Second, it is only applicable to fresh tissue samples. For frozen samples, since cells have lost their activity, scRNA-seq cannot be performed. This substantially restricts its application, increases the difficulty of operation and reduces the sample flux. For example, in order to ensure the stability of RNA, many clinical samples need to be frozen. Such archived frozen samples are no longer amenable to scRNA-seq and their value could not be fully tapped by the latest technology (73). Third, the dissociation process induces the expression of stress genes, resulting in “artificial transcriptional stress responses” of cell transcription and eventually to transcription bias. The data so obtained cannot truly reflect the cell transcription status of the sample, and the reliability of the results is greatly impaired. This has been demonstrated by a great many experiments. For example, Brink et al. found that the process of protease dissociation at 37°C would induce the expression of stress genes, thereby introducing human errors, and leading to inaccurate results of cell type identification (74). The latest comparative experiment further confirmed this limitation: Dissociation at 37°C induced an increased expression of multiple stress genes, which yielded seriously distorted results, and the low-temperature dissociation could effectively avoid this phenomenon (75). Fourth, for many solid tissues, such as cerebral, cardiac and renal tissues, protease tends to dissociate the cell types that are subject to dissociation, thus losing the cells that are not easy to dissociate. At the same time, some sensitive cells may be damaged due to excessive dissociation. Therefore, the dissociation process cannot effectively obtain all cell types in the tissue, and the accuracy of the results is substantially affected (76–79). It is believed that, with the continuous improvement and breakthroughs of the technology, single-cell sequencing technology has a good prospect of being widely used in the research and treatment of lung cancer.

### 2.2 Proteome

#### 2.2.1 single-cell proteomic analysis

Single-cell proteomics allows for analysis of protein expression at the single-cell level, thus revealing fine differences between individual cells. It provides a powerful tool for the analysis of cell and tumor heterogeneity, specific cell types, circulating tumor cells (CTC), immunological research, the genetic study of cell cycle, and the examination of trace/rare samples. Working on different principles and depending on various scenarios, many methods for the quantitative detection of single-cell proteins have emerged in recent years, including microfluidic techniques, microporous methods, optical fiber nano biosensoring, fluorescent probing and mass spectrometry-based single-cell protein detection (80, 81). Especially in recent years, with the rapid development of mass spectrometry technology, the bottleneck of proteomic research based on mass spectrometry has been removed, and the scanning speed and detection sensitivity have been greatly improved, which makes it possible to detect extremely trace protein samples.

#### 2.2.2 Mass spectrometry flow cytometry

CyTOF is a flow cytometry technique based on mass spectrometry principles and is used for multi-parameter detection of individual cells. By integrating mass spectrometry and traditional flow cytometry, CyTOF not only keeps the high-speed of traditional flow cytometry, but also attains the high-resolution of mass spectrometry, overcomes the problem of overlapping light spectra of traditional flow cytometry fluorescence emission groups, and is able to simultaneously detect more than 30 protein markers in tumor cells. This high-dimensional single-cell technique is described as the “single-cell atlas” of the tumor ecosystem. As a single-cell high-dimensional immune analysis, it can better link the tumor immunity map with its clinicopathological characteristics (82, 83).

Lavin Y et al. (33) separated immune cells from tumor tissue, normal lung tissue and peripheral blood from lung cancer patients, detected specific transcripts of cells and more than 30 protein markers on the surface using CyTOF and other technologies, and drew a detailed map of immune cells in the TME of early-stage lung adenocarcinoma, to provide an experimental basis for the design of immunotherapeutic regimen for early lung cancer. The unsupervised cluster analysis of the three tissues divided T cells into 21 subgroups in terms of different surface marker expression patterns, including new subgroups (IX, XX, etc.) that had not been previously identified by traditional methods. In addition, the analysis of the proportion of subpopulations in different samples showed that the content of Treg cells in early-stage tumor patients was significantly increased, and it grew rapidly at the early stage of tumor, and PD-1 expression was significantly elevated in CD4+ and CD8+ T cells in tumor tissue.

Karacosta LG et al. (36) used CyTOF to identify and characterize the epithelial mesenchymal transformation (EMT) state of clinical lung cancer specimens according to the immune state map obtained in lung cancer cell lines. The researchers used HCC827 cell line for study of EMT, and examined 28 protein expression markers to characterize the EMT status and proliferation-, signal transduction- and apoptosis-related status by employing qualitative CyTOF. Their study observed an increased expression of PD-L1 during EMT, confirming that EMT was related to tumor immune escape. Notably, the researchers found that, among the EMT transcription factors, Oct3/4 and Nanog expression was significantly up-regulated during the entire EMT process, indicating that the co-expression of Oct3/4 and Nanog was crucial to the EMT of lung cancer cell lines. Shaul ME et al. (37) clinically assessed the level of circulating high-density neutrophils (HDN) and low-density neutrophils (LDN) in patients with advanced lung cancer by using CyToF, and found that the three main subtypes of LDN/HDN possessed immune characteristics and inherent plasticity. These findings laid foundation to the development of new tumor immunotherapies.

#### 2.2.3 Imaging mass cytometry

Imaging mass spectrometry (IMC) combines high-resolution imaging technology and CyTOF technology to generate tissue structure images involving multiple factors such as cell markers, transcripts, and transduction signals, so as to achieve single-cell proteome spatial analysis (84). In the field of immune oncology, IMC can classify infiltrating immune cells in a high parameter space while maintaining its spatial coordinates, which may provide useful information about host responses and inform the selection of appropriate immunotherapies (85).

Sorin M et al. (38) used IMC to describe histopathological patterns of pulmonary adenocarcinoma and the immune cell landscape in 416 patients, and analyzed more than 1.6 million cells, and conducted spatial analyses on immune cell lineages and activation status with different clinical relevance (including survival). Their analyses on the category of cellular neighborhood and survival time confirmed the association between specific cell interactions and survival rate, indicating that the tissue relationship of cells in the immune microenvironment is of uniquely prognostic value. They also studied the relationship between cell phenotype and survival in the TME. The results showed that the total number of neutrophils exerted no significant impact on survival, but the increased proportion of HIF1α+ subgroup was significantly correlated with poor overall survival.

Sorin M and their colleagues (39) also performed IMC on 114,524 single cells from 27 NSCLC patients receiving ICI, and achieved spatial resolution of immune spectrum and activation state with different clinical results. These studies proved that the expression of CXCL13 was related to the ICI efficacy, and the recombinant CXCL13 enhanced the response to anti-PD-1 *in vivo*, which could be ascribed to increased T-cell subpopulations subjected to antigen stimulation and decreased CCR2+ monocytes. These findings highlighted the importance of major immune cell lineages and their functional states in the response to ICIs and help us better understand the role of the tumor immune microenvironment in such response.

These observations highlighted the importance of evaluating immune cell phenotypes at the single-cell level. In fact, both CyTOF and IMC are particularly useful methods for characterizing the specific phenotypes of cells involved in responses to immunotherapy at the single-cell level. With immunotherapy, single-cell proteomic analysis can provide insights into the signaling pathways related to the effectiveness of immunotherapies and drug resistance. At the entrance into the era of single-cell proteomics, we are still faced with great challenges in proteome coverage depth and flux. We believe that these challenges can be addressed by integrating mass spectrometry flow cytometry, measurement strategies, and algorithms.

### 2.3 Genome

Single-cell genome sequencing is a new technique that sequences and amplifies the entire genome within a single cell (86). A complete genome with high coverage can be obtained by efficient amplification of a small amount of whole-genome DNA from isolated single cells, followed by high-throughput sequencing. In fact, single-cell genome sequencing has become a powerful tool for study of the heterogeneity between cells in biological samples and identification of genomic changes (such as copy number variation and point mutation). This technology presents unique advantages in the research of cell lineage differentiation, especially cell evolution during tumorigenesis, and cell heterogeneity in complex biological samples (87, 88).

In recent years, many single-cell genome amplification technologies have been developed, such as DOP-PCR, multiple annealing, multiple displacement amplification (MDA) and loop-based amplification cycle (MALBAC), transposon insertion-mediated linear amplification (LIANTI), etc. (89). Single-cell whole exome and whole genome sequencing techniques have been developed (90). Although they have not been used for the study of lung cancer immunotherapies, single-cell genome sequencing has been employed for the analysis of a large number of single cells. In the entire process of lung cancer management, we can track the specific gene variation of lung cancer patients and the heterogeneity of tumor cell population clonal evolution (91–93), which suggests that this technology has a good prospect of being applied in the research of lung cancer immunotherapies.

The single-cell genome sequencing of single circulating tumor cells (CTC) shows some unique advantages in the diagnosis, differential diagnosis, monitoring and prognosis of lung cancer. For example, Su Z et al. (91) conducted single-cell whole genome sequencing on CTC from 48 SCLC patients, and compared the sequence data with the mutations in tumor tissue of the same patient. They found that most gene mutations in tumor tissue could be accurately detected in CTC, and DNA had conspicuous heterogeneity, suggesting that single-cell genome detection of CTC is an effective way to monitor the genetic variation and disease progression of SCLC. Ni X et al. (92) used MALBAC technology to sequence the whole exome and genome of a single cell in CTC of lung cancer patients, and found a new copy number variation profile. By detecting the copy number change (CNV) of CTC, tumor metastasis could be monitored. The research team further analyzed the CNV profile of individual CTC from 11 patients with different subtypes of lung cancer, and identified the different lung cancer subtypes, indicating that it is feasible to use the CNV analysis of CTC to classify tumors in the future. Chen J et al. (93) found, by using the dimensional analysis of the single-cell genome, that different driver changes and the initial EGFR mutation co-existed in the same cancer cell in the patients with Osimertinib-resistant NSCLC. The heterogeneity of clonal evolution of tumor cell populations led to the development of Osimertinib resistance. However, at present, no literature reported single-cell genome sequencing for the prognostic prediction of immunotherapy for lung cancer. Further studies are needed in this field.

### 2.4 Epigenomics

Single-cell epigenomics allows for the analysis of information about chromatin modifications and their potential regulatory effects at single-cell resolution, and can complement data beyond RNA expression and DNA variation obtained by single-cell DNA sequencing and RNA sequencing (94, 95). Combination of single-cell epigenomics with single-cell transcriptomics can help us better understand the cell type-specific gene regulation program and how the tumor cells change in response to environmental stimuli (96–98). These subjects are the important directions of future studies on single-cell analysis in lung cancer immunotherapy. Epigenomic analysis has been used for single-cell study, such as ATAC-seq, ChIP-seq, bisulfite-based DNA methylation sequencing, and chromosome conformation capture techniques (3C and Hi-C) (99–101). Among these techniques, single-cell ATAC-seq (scATAC-seq) is currently the only widely used method with sufficiently high throughput, being capable of detecting the openness of chromatin in different cells at the single-cell level and showing the sites of different transcription factors and regulatory factors. Although it has not been used in the study of lung cancer immunotherapy, it has found widespread application in the research of lung cancer heterogeneity, tumor microenvironment and other fields.

In order to gain insights into the intratumoral heterogeneity of lung squamous cell carcinoma, Wang et al. (102) performed single-cell ATAC seq on an LUSC patient, and detected a total of 50486 peaks. The open chromatin map was highly consistent with the bulk NSCLC sample. On the level of single-cell analysis, high heterogeneity was observed in some open chromatin regions. LaFave LM et al. (103) used single-cell epigenomics to analyze the chromatin state transition in the mouse model of LUAD, and identified a pre-metastasis transition in lung adenocarcinoma, characterized by the activation of the RUNX transcription factor, which mediates the remodeling of extracellular matrix to facilitate metastasis, and is indicative of the survival rate of LUAD patients. Their findings proved that the single-cell epigenomics plays an important role in the identification of regulatory programs and can help reveal the mechanism of tumor progression and key biomarkers.

Compared with scRNA-seq, one of the main advantages of scATAC-seq lies in that it can provide more in-depth understanding of gene regulation and transcription or other processes, and more information about cell lineages and characteristics. However, the scATAC-seq is still restricted by some technological limitations, including limited data and high sensitivity to tissue separation. In addition, no literature covered the application of single-cell epigenome in lung cancer immunotherapy, and further studies are warranted in the future.

### 2.5 multimodal omics analysis

Single-cell multiomics refers to the cutting-edge technology of measuring multiple omics data simultaneously in the same cell (18, 104). For example, the recently developed CITE-seq (105) technology is designed to couple specific oligonucleotides to different antibodies, so that it can convert the measurement of proteins into the measurement of DNA tags (ADTs) connected to antibodies. Therefore, CITE-seq can determine the abundance of RNA and cell surface protein in the same cell by sequencing. In addition, with the progress of new technologies, transcriptome has been able to be used simultaneously with other genomics at the level of single-cell analysis, including ATAC (97, 106), DNA methylation (107), nucleosome distribution (108), spatial location (109, 110), among others, which overcomes the inherent limitations of scRNA-seq and helps researchers further understand how other genomics affect the state and function of cells (18). The utilization of single-cell multiomics technology in immune oncology can identify the heterogeneity of immune cells in tumors and reveal the interaction and mode among multiple cell groups in the process of differentiation, so that researchers can look into the role of immune cells in the growth of tumor, so it has significant application prospects in immunotherapy research (111).

In order to study the molecular state and composition of immune cells in NSCLC, Leader AM et al. (40) carried out single-cell analysis of NSCLC by using scRNA-seq, CITE-seq and TCR-seq (T cell receptor sequencing), and constructed the immunoreactive cell atlas of early lung cancer, and established the LCAM module for detailed classification of NSCLC tumors by analyzing immune cell types. The high score of LCAM indicates that the patient was undergoing a stronger antigen-specific anti-tumor adaptive immune response. Therefore, LCAM can serve as a more direct indicator of the activation of antigen-specific anti-tumor immunity, providing important reference data for the selection of targets for immunotherapy. Hanada KI et al. (41) used CITE-seq and TCR-seq to analyze tumor-infiltrating lymphocytes (TILs) in NSCLC, and defined a new antigen-reactive T cell molecular tag in terms of the expression of CD39 protein and CXCL13 mRNA to efficiently identify CD4+and CD8+T cells with new antigen-reactive TCRs. Zhang L et al. (112) showed the unique cell composition and gene expression profile of LUAD and LUSC through the multi-group analysis on the basis of single-cell transcriptome, which provided insights into the pathogenesis and heterogeneity formation of various types of lung cancer. At the same time, several highly-expressed genes identified in early lung cancer samples can provide clues to potential targets for early treatment of lung cancer.

Single-cell multimodal omics aims to integrate multiple molecular information from the same single cell (such as at DNA and RNA, RNA and ATAC levels) or at all three levels. These methods can provide more insights into genotype phenotype relationship and epigenomic regulation of gene expression. Although the initial result has proven the feasibility of sequencing DNA and RNA in the same cell, currently, their throughput remains low and the cost is high. The future development of these methods using nanopore systems, droplet platforms, and combinatorial indexing is expected to overcome many of these technical barriers, thereby expanding their application in lung cancer research.

### 2.6 Single-cell TCR analysis

In our immune system, T cells play an important part in the acquired immune response. T cell receptor (TCR) is a protein on the surface of T cells responsible for specific recognition and binding with major histocompatibility complex (MHC) antigen peptides (113). In tumor tissue, when TCR on tumor-infiltrating T cells recognizes and binds to tumor antigen-MHC complex, T lymphocytes are activated *via* signal transduction and enter the subsequent immune response process, which enables immunotherapy to effectively elicit antigen-specific anti-tumor immune response (114). TCR sequencing targeting tissue or population cells can reflect the expression status of cell groups to a certain extent, but cannot determine the state of specific cells in a certain cell group. With the rapid development of single-cell sequencing techniques, TCR sequencing has also advanced from bulk TCR sequencing to single-cell TCR sequencing (scTCR-seq). ScTCR-seq is a high-throughput sequencing technique for detecting TCR molecular sequences at the level of single-cell analysis, and can provide information on the role of TCR sequences in T cells-specific selection, activation, and phenotypic identification, as well as T cell differentiation pathways. ScTCR-seq can achieve higher cell-processing throughput and accuracy, so immunophenotypic analysis at the level of single-cell analysis is increasingly used in immunological research (115–117).

In a study on lung cancer immunotherapy, Ma Yd et al. (42) developed a scTCR-seq technology based on RNA pre-amplification, and used this technology to identify tumor-specific TCR from lung cancer-specific TILs at high efficiency and low cost. Further functional verification showed that its corresponding TCR-T cells could specifically recognize and kill autotumor cells, which can be potentially used for individualized immunotherapy for advanced lung cancer. Ott PA et al. (43), in their clinical trials, used personalized tumor neoantigen vaccine (NEO-PV-01) and PD-1 inhibitor in the treatment of three kinds of high TMB, metastatic tumors (NSCLC, advanced bladder and melanoma cancer) for the first time, and the scTCR-seq analysis revealed the dynamic changes in the clonal type of tumor neoantigen vaccine-specific T cells, and accurately detailed characterized the T cell response. From the cellular level, they proved that the T cell immune response induced by tumor new antigen vaccine was highly specific and effective. Hui Z et al. (44) conducted scRNA-seq and scTCR-seq analyses on the immune cells from NSCLC patients who had received neoadjuvant immunotherapy but not immunotherapy. They found that the enrichment of B cells and CD4+T cells was related to the more favorable prognosis in NSCLC patients. IL-21 was essential for tumor control and the transformation of the B-cells to anti-tumor IgG1 and IgG3 subtypes. In addition, TNFRSF4 can potentially be used as a molecular target to reduce the function of Treg and improve the anti-tumor immunity against NSCLC, which help us better understand the mechanism of cell synergy in the clinical response to neoadjuvant immunotherapy.

It is of importance to have an in-depth understanding of the clonal dynamics and functional status of T cells in NSCLC to improve the efficacy of immunotherapy. Zhang F et al. (45) conducted scRNA-seq and scTCR-seq on T cells from the peripheral blood of NSCLC patients before and after PD-1 blockade, identified single peripheral T cell clones, and dynamically monitored their changes during immunotherapy. They found that tumor-related CD4+T cell clones had higher cytotoxicity than their CD8+T counterparts. When lung cancer progresses, the number of tumor-related CD4+T cell clones decreased significantly, and the proportion of PD-1+T cells dropped. In addition, the pseudo-time track of CD8+T cell clone corresponded to the treatment time point, indicating that the “cytokine receptor-cytokine interaction” pathway was down-regulated. These analyses help us better understand the dynamics of T cell clones from the peripheral blood of NSCLC patients during PD-1 blockade. To study cloning relationship between NSCLC single T cells, Guo X et al. (27), again, used scTCR-seq and obtained, in 16 cell clusters, 8038 full-length TCRs containing both α-chain and β-chain. Of them, 5015 cells had unique TCRs, and 3023 cells repeatedly used TCRs, indicating that the clones were expanding. The state transition of CD8+T cell clusters in NSCLC was deduced by detecting TCRs, including the inherent T cell development and tumor-induced T cell exhaustion. By using single-cell RNA sequencing and TCR sequencing, Gueguen P et al. (46) found two CD8+TIL sub-populations in NSCLC that expressed memory-like gene modules. The differentiation of these two sub-populations from precursor to late stage was found to be related to TCR amplification and T-cell cycle in tumor. These findings provided important evidence regarding the origin, ontogenesis and functional organization of TIL in NSCLC.

In summary, scTCR-seq adds key information about the antigenic specificity of T cells to immune cell analysis, enabling a more refined dissection of the role of antigenic specific T cells in the response to immunotherapy. What is more, non-invasive identification of amplified TCR clones in vertically collected blood samples during lung cancer immunotherapy can accurately characterize the immune activity of T cell subpopulations related to treatment response, making immunotherapy monitoring more accurate.

## 3 High dimensional space analysis

The spatial cell composition of tumors is inconsistent. The spatial distribution of tumor subclones and the spatial variability of immune microenvironment are believed to be responsible for the heterogeneity of most cancer types and the variability of immunotherapy response (118–120). Single-cell space technology includes image-based spatial proteomics technology in combination with analyses of single-cell resolution, and variation information at DNA level and changes in RNA level expression, and multi-dimensionally analyzes research objectives (121). By quantitatively determining tens to hundreds of genes, transcripts or proteins, single-cell space technology can garner valuable molecular, cellular and micro-environmental information under the background of cell structure, and help researchers to look into the interaction between cells, the relationship between tumor cells and TME, and patients’ response to immunotherapy from the perspective of cell spatial configuration (122, 123).

Because obtaining single-cell suspension entails enzymatic hydrolysis of tissues, the single-cell transcriptomic sequencing leads to loss of the spatial location information of cells during tissue lysis, and the spatial information is crucial for the understanding of the cell microenvironment and the interaction between cells (124). Single-cell space transcriptome technology (121) overcomes the limitations of scRNA-seq, and can combine gene expression with the immunohistochemical image of tissue samples, thereby locating the gene expression information of various cells in the tissue in terms of the original spatial location of the tissue, identify genes that are active in the tissue, and can visually detect the gene expression difference in various parts of the tissue. Single-cell space transcriptome technology has been widely used in the study of spatial distribution of cancer cells and TME, which are important to the understanding of the relationship between tumor heterogeneity and TME (125, 126).

Zhu J et al. (47) used single-cell transcriptome and spatial transcriptomic technology to map the changes of cell heterogeneity and spatial distribution in the progression from adenocarcinoma *in situ* to microinvasive adenocarcinoma and further to invasive adenocarcinoma, and found that, with LUAD progressing from adenocarcinoma *in situ* to invasive adenocarcinoma, the spatial distribution of cancer cells became increasingly evident, and the malignant features of the tumor margin became more conspicuous, while UBE2C+cancer cell subgroup played a crucial role in promoting this process. The results of single-cell space transcriptome showed that, in adenocarcinoma in situ, there was no Treg in the cancer area, while in invasive adenocarcinoma, cancer cells recruited Treg to the cancerous regions, suggesting that Treg accumulation in the cancer area initiated the invasion process of LUAD and that TGF-β signaling pathways are involved in cancer cell interaction with the TME and spatial changes in regulating immune escape in the invasion of LUAD. The crosstalk between the components of TME impacts the tumor progression largely by mediating the immunosuppressive phenotype. Sinjab et al. (127) found that the overlap of immune checkpoint-receptor and cytokine receptor (L-R) interactions between LUAD tumor-distal regions was reduced compared with L-R interactions between LUAD tumor-proximal regions (including adjacent tissues and moderately distant tissues). It is noteworthy that, in the samples of multiple patients, the interaction between the immune checkpoint proteins CD24 and LGALS9 in tumor epithelial cells, SIGLEC10 in dendritic cells, SIGLEC10 and HAVCR2 in macrophages increased. These intercellular interactions exhibited differential enrichment in LUAD tumor tissues and LUAD normal tissues. These findings suggested that the LUAD ecosystem had intercellular communication that promotes tumor inflammation and enhances immunosuppressive states.

Visual imaging of proteins is usually achieved by the fusion expression of antibodies or fluorescent proteins. Currently, image-based single-cell spatial proteomics improves the multiple capabilities of proteomic spatial analysis of up to nearly 100 markers, thereby expanding the number of representable cell states and cell types, and providing an opportunity to visualize and study proteins in the cellular environment (128, 129). Growing studies have shown that cell populations with the same genetic background also present differences in protein expression and protein location. The image-based spatial proteomic technology is helpful to the study of this variability since it captures the protein spatial distribution at single-cell resolution, so as to obtain the protein characteristic expression spectrum of tissues of different regions, and it is widely used in the studies of tumor cell heterogeneity, which is of great value for the analysis of TME, diagnosis and prognosis (130, 131).

Zugazagoutia J et al. (48) performed DSP spatial proteomic analysis and multiplexed immunofluorescence (mIF) detection on FFPE samples in the form of tissue microarray (TMA) in 53 patients with advanced NSCLC who had received PD-1 checkpoint inhibitor monotherapy. They demonstrated that the high-level CD56 expression in the immune cell region (CD45+) was associated with longer PFS and OS in NSCLC patients receiving PD-1 checkpoint inhibitor monotherapy. Moutafi MK et al. (49) analyzed the spatial *in situ* expression data of 71 proteins in NSCLC samples by using DSP technology, and found that the expression of CD44 in tumor cells was closely related to the prolonged OS and PFS, which can be used as an independent factor for the prediction of the clinical efficacy in patients receiving PD-1 inhibitor treatment. However, for patients without receiving immunotherapy, the high expression of CD44 in tumor cells had no prognostic effect. In addition, researchers also found that a unique immune microenvironment developed in the tumor region whose tumor cells had high expression of CD44, suggesting that the expression of an array of immunoregulatory molecules, such as PD-L1, TIM-3, ICOS and CD40, was significantly up-regulated. It showed that the expression of CD44 in lung cancer cells can function as a new independent biomarker that supplements the existing biomarkers for optimal patient stratification, and may open up a new and better immunotherapeutic strategy for lung cancer.

Although single-cell space technology remains at its early stages, spatially resolved multiplex analyses are reshaping our way to look at cellular interactions and structural relationships between tumor cells and TME cells, which affect tumor immunity and dictate patients’ response to immunotherapy. Moreover, spatial localization multiple techniques can add genotypic and phenotypic dimensions to our understanding of cell interactions in the tumor immune microenvironment, and represents the next frontier in the elucidation of the mechanism underlying the resistance of lung cancer to immunotherapy. Therefore, clinical application of single-cell space technology can help us better understand the tumor response and resistance to immunotherapy.

## 4 Conclusion

Single-cell techniques have revolutionized our way to look at complex diseases, such as lung cancer, providing unprecedented insights into the heterogeneity of tumor cells and the tumor microenvironment. In fact, these techniques help us better understand lung cancer and its response to immunotherapy, and thereby develop more efficacious therapeutic strategies.

1. **In-depth Understanding of Tumor Heterogeneity:** By studying tumor heterogeneity at a single-cell level, researchers can identify subpopulations of cells that drive resistance to treatment, leading to the development of more personalized therapies.
2. **Characterizing Tumor-Immune Interactions:** Single-cell techniques enable the profiling of both tumor and immune cells simultaneously, shedding light on the communications and signaling pathways involved in the immune evasion and tumor progression.
3. **Discovery of Novel Biomarkers:** Single-cell analyses can identify rare cell populations or immune cell subsets that are specifically implicated in the modulation of the tumor immune response. These new biomarkers can be validated and used for patient selection.
4. **Uncovering Mechanisms Underlying Resistance:** Single-cell techniques help researchers look into the cellular and molecular mechanisms that underlie drug resistance, help us understand how tumors evolve and escape immune surveillance. This knowledge can inform the development of combination therapies that overcome resistance.
5. **Targeting Tumor-Resident Immune Cells:** Single-cell techniques aid in the identification of tumor-resident immune cells and their functional features. By targeting these cells, researchers can work out therapies to reprogram the immune response and thereby enhance anti-tumor immunity.
6. **Treatment Monitoring and Precision Medicine:** Single-cell techniques can be applied to the analysis of liquid biopsies (e.g., circulating tumor cells or cell-free DNA) from patients. This allows for non-invasive monitoring of treatment response and disease progression, informing treatment decisions and the adjustment of therapies in real-time manner to achieve precision medicine.

## Author contributions

PL, QH, JZ are equal contribution and first authorship. QHH, JL, LZ are correspondence. The authors declare that they have no competing interests.
